# The Role of Megakaryocyte Assessment in Bone Marrow Cytology

**DOI:** 10.3390/jcm14082681

**Published:** 2025-04-14

**Authors:** Monika Błocka-Gumowska, Iwona Hus, Agnieszka Szymczyk

**Affiliations:** 1 Laboratory Diagnostics Centre, National Medical Institute of the Ministry of Interior and Administration, Wołoska 137 Str., 02-507 Warsaw, Poland; monika.blockagumowska@wp.pl; 2 Department of Hematology, National Medical Institute of the Ministry of Interior and Administration, Wołoska 137 Str., 02-507 Warsaw, Poland; iwonach.hus@gmail.com

**Keywords:** megakaryocyte, bone marrow cytology, megakaryopoiesis, dysmegakaryopoiesis

## Abstract

Despite the progress made in recent years in hematological diagnostics, cytological assessment of bone marrow, including the assessment of megakaryocytes, is still an important element of the diagnostic process. It is one of the most accurate methods for detecting even minor abnormalities, which is often superior to histopathological examination in this respect. Although, according to current recommendations, megakaryocytes are not included in the total percentage formula of the myelogram, the assessment of platelet function, morphological features, and quantitative disorders allows for the direction of further diagnostic process. In this article, we present the principles of assessing megakaryocytes in a bone marrow aspirate smear. We also characterize normal megakaryocytes at various stages of megakaryopoiesis, atypical megakaryocytes in pathological and physiological conditions, and cells with a similar morphology to megakaryocytes.

## 1. Introduction

In recent years, knowledge about the regulation of megakaryocyte maturation and platelet formation has significantly progressed [1,2,3,4]. The image of megakaryocytes has evolved from single static vascular cells located within bone marrow vascular districts to dynamic maturing cells that may change their location under the influence of specific stimuli [5,6,7].

Megakaryocytes are cells that have been identified not only in the bone marrow and peripheral blood but also in the fetal liver, yolk sac, spleen, lungs, and circulatory system. Recent studies confirm that they are responsible not only for platelet production but also for modulating the immune response and can affect the bone marrow stromal niche [6,8,9].

Despite the progress that has been made, in everyday clinical practice, cytological assessment of the bone marrow, including the assessment of megakaryocytes, is still an important element of the diagnostic process. Megakaryocyte aberrations are most pronounced in myeloproliferative neoplasms, but quantitative and qualitative abnormalities are found not only in hematological diseases but also in general internal diseases or some physiological conditions [10,11,12]. In this article, we characterize normal megakaryocytes at various stages of maturation, the causes of qualitative and quantitative abnormalities, and the morphology of these cells in various pathological conditions.

## 2. Materials and Methods

For this review article, bone marrow smears from patients diagnosed at the Department of Hematology, National Medical Institute of the Ministry of Interior and Administration, in which the cytomorphology of megakaryocytes correlated with the clinical and cytogenetic presentation were selected. Bone marrow smear preparation was performed with a uniform procedure according to the recommendations by the International Council for Standardization in Haematology (ICSH). Smears were prepared from samples without an anticoagulant. The marrow area on every smear was approximately 1.3–1.5 × 3.0 cm with proper and relatively uniform thickness. The smears were air-dried and stained using the May-Grünwald Giemsa (MGG) method. Megakaryocytes were searched for using the lowest magnification (10×), also examining the edges and ends of the slides. In cases with dysplasia, ≥30 megakaryocytes were evaluated, and the frequency of morphological abnormalities was recorded under 40× or 100× magnification. For differential staining of blood and bone marrow cellular elements, we used May-Gruwald-Giemsa stains (manufacturer “Aqua-Med”ZPAM-Kolasa, Łódź, Poland) and phosphate buffer pH 6.8 (manufacturer “Chempur”, Piekary Śląskie, Poland). Megakaryocyte images were taken using a Leica DM2000 microscope and a Leica DMC2600 camera (manufacturer KAWA.SKA, LLC, Zalesie Górne, Poland). In this article, we present photos that have not been published anywhere before.

## 3. Megakaryopoiesis

Megakaryocytes are derived from hematopoietic stem cells (HSCs) and constitute <0.05% of all nucleated bone marrow cells [4,6]. In normal bone marrow, they occur near the sinuses, at a distance from the bone trabeculae, and do not form clusters larger than 2–3 cells [10,12]. The sites of megakaryopoiesis change during embryonic and fetal development, and megakaryocytes in the liver and circulatory system are detected as early as 8 weeks after fertilization [5,7]. Megakaryopoiesis occurring in the bone marrow is a multi-stage and complex process that begins with a hematopoietic stem cell (HSC) through the early progenitor cell stage (granulocyte, erythroid, macrophage, and megakaryocyte colony-forming cells; GEMM-CFCs), directed cells, i.e., multipotent progenitor cells (burst-forming unit megakaryocyte; BFU-Mk), and megakaryocyte progenitor cells (colony-forming unit megakaryocyte cell; CFU-Mk) to the formation of an immature and then mature megakaryocyte [13]. The proper process of differentiation and maturation of megakaryocytes requires the participation of numerous cytokines and growth factors, such as TPO (thrombopoietin, the main physiological regulator of platelet production) [14], G-CSF (granulocyte-colony stimulating factor), EPO (erythropoietin) [15], IL-3 (interleukin-3), IL-6 interleukin-6), IL-11 (interleukin-11), and KL (C-kit ligand); transcription factors, such as SCL/TAL1 (stem cell leukemia/T-cell acute lymphoblastic leukemia [T-ALL] 1), GATA1 (globin transcription factor 1), GATA2 (globin transcription factor 2), RUNX1 (Runt-related transcription factor 1), FLI1 (friend leukemia integration), and GFI-1b (growth factor independent 1B transcriptional repressor); and chemokines, including SDF-1 (stromal cell-derived factor 1) and transmembrane protein Notch [4,6,8,9,10,12,16]. At the final stage of maturation, platelets are produced [4,8,9,12,17]. Platelets, also called thrombocytes, are anucleate fragments of the megakaryocyte cytoplasm, playing an important role in the processes of primary and secondary hemostasis. In an adult, approximately 1 × 10^11^ platelets are produced every day, and in the period of increased demand, their number can increase even 10-fold [4,6].

## 4. Morphology of Normal Megakaryocytes

Different classification schemes are used to determine the stages of megakaryocyte maturation, which are based on morphological differences in the nucleus (N), cytoplasm (C), and the N/C ratio. Based on this features, we can distinguish three characteristic stages of megakaryocyte maturation [4,6].

**Figure 1:**
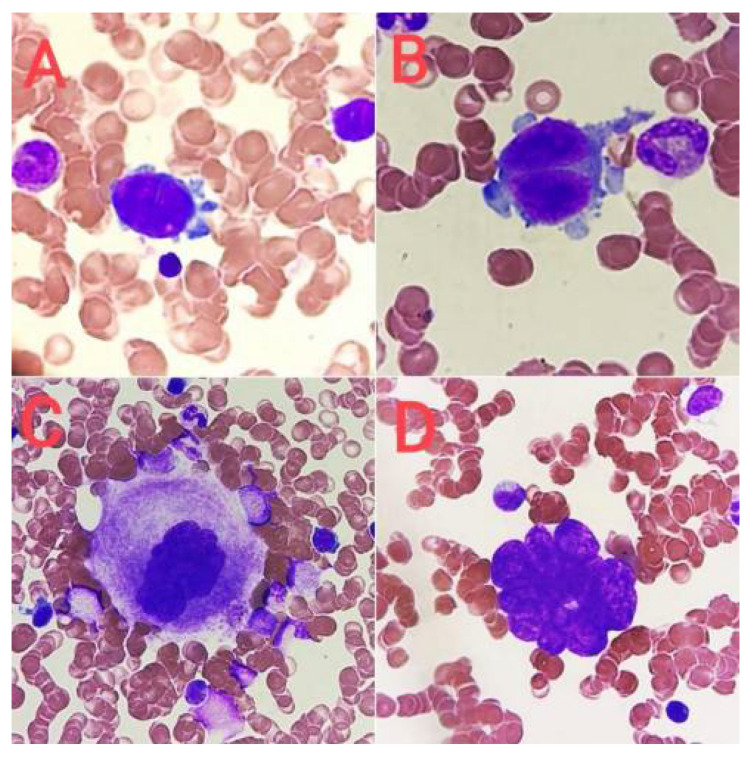
Stages of megakaryocyte maturation. (**A**) Megakaryoblast, (**B**) promegakaryocyte, (**C**) mature megakaryocyte, and (**D**) naked megakaryocyte nucleus.

Stage I corresponds morphologically to the megakaryoblast. Megakaryoblasts constitute about 25% of all cells of the platelet-forming lineage [6]. The cell diameter is similar to that of the myeloblast and ranges from 6 to 24 µm (average about 15 µm). The nucleus is round and minimally slightly indented (2–4 N), the chromatin is delicate, and nucleoli are visible. These cells are characterized by a high nucleus/cytoplasm ratio (N/C). The cytoplasm is basophilic and sparse and often occurs in the form of vesicles at the edges of the cell (Figure 1A). Megakaryoblasts do not produce platelets [10,12].

Stage II corresponds to promegakaryocytes. These cells, like megakaryoblasts, constitute about 25% of all cells of the megakaryocytic lineage [6]. Their diameter is about 20 μm. They show marked nuclear lobulation, chromatin condensation features, and a moderate N/C ratio. The cytoplasm is basophilic or polychromatophilic and granular or with slight pink granulation [10,12] (Figure 1B).

Stage III is a mature megakaryocyte, which is a cell with a diameter of 25 to 64 μm with a single multilobed nucleus (usually from three to seven lobes). It is characterized by a low N/C ratio and DNA content from 4 N to 64 N. The cytoplasm is abundant, with a pale pink to pink color, with numerous azurophilic granules or clusters of granules and visible proplatelet demarcations [4,10,12]. Platelets are visible around the cell (Figure 1C). Some authors divide mature megakaryocytes into early and late (stage III and stage IV) [18,19].

The group of mature cells also includes naked megakaryocyte nuclei (Figure 1D), which arise as a result of complete fragmentation of the cell membrane during platelet production. Mature megakaryocytes constitute about 50% of all cells of the platelet-forming lineage [4,6]. The entire maturation process from megakaryoblast to mature megakaryocyte lasts about 72 h [4,6].

## 5. Megakaryocyte Differentiation and Maturation

The differentiation of megakaryocyte lineage cells consists of the proliferation of mononuclear progenitor cells, followed by endoreplication or endomitosis of precursor cells, which is a controlled process of DNA replication with no cell division. It begins at the final stage of the megakaryoblast. It results in the formation of ploidy megakaryocytes (usually from 8 N to 64 N) with an increased cytoplasm volume [6,20]. Higher or lower ploidy most often accompanies various pathological conditions. Under normal conditions, the number of endomitotic cycles ranges from two to five, but most megakaryocytes undergo three cycles of endomitosis and reach a DNA content of 16 N [10,11,21].

After the end of endomitosis, polyploid megakaryocytes begin the maturation process, which involves granules, a dense network of channels, and a network of so-called demarcation membranes, which are the most characteristic features of megakaryocyte maturity [10,11,22].

## 6. Megakaryocytes in Peripheral Blood

Megakaryocytes migrate through the bone marrow endothelial clefts into the circulation, where they are captured in the pulmonary capillaries. Megakaryocytes (usually in the form of naked nuclei) are occasionally found in the peripheral blood of healthy individuals [6,11].

**Figure 2:**
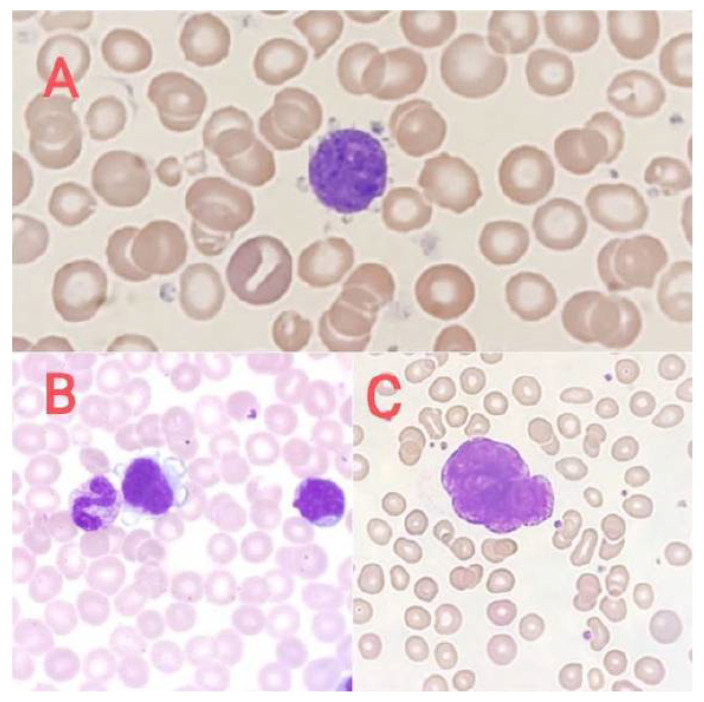
Megakaryocytes in peripheral blood. (**A**) Micromegakaryocyte in blood smear, (**B**) megakaryoblast in blood smear, and (**C**) naked megakaryocyte nucleus in blood smear.

Naked nuclei of megakaryocytes (Figure 2C) are most often described in peripheral blood smears in newborns, in women in the postpartum period, or in patients after surgery [23,24]. In pathological conditions, atypical megakaryocytes are found in blood smears, in primary myelofibrosis (micromegakaryocytes, Figure 2A), and in acute megakaryoblastic leukemia and other myeloproliferative neoplasms (megakaryoblasts, Figure 2B), among others.

## 7. Quantitative and Qualitative Changes

**Figure 3:**
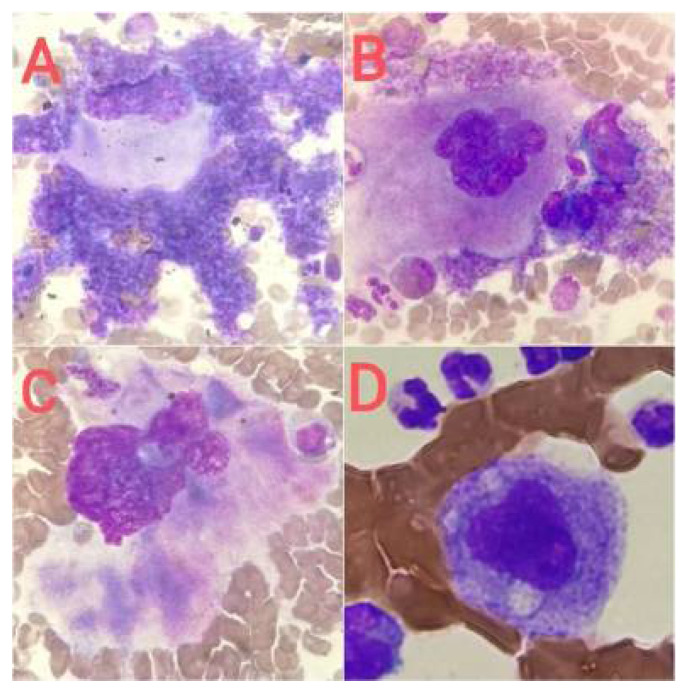
Reactive changes in megakaryocytes. (**A**) Megakaryocyte showing increased platelet-forming activity, (**B**) megakaryocyte cluster (mature form and two younger forms), (**C**) cytoplasm maturation disorders, and (**D**) megakaryocyte cytoplasm vacuolation.

**Figure 4:**
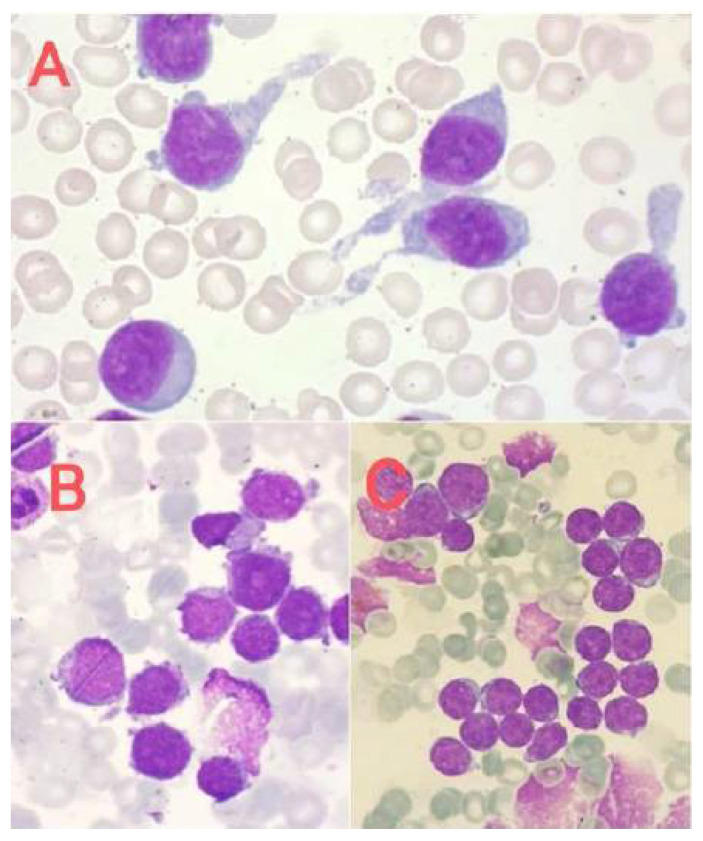
Acute megakaryoblastic leukemia. (**A**) Megakaryoblasts with cytoplasm in the form of elongated, caudate processes, (**B**) megacryoblasts with visible nucleoli and basophilic cytoplasm forming small processes, and (**C**) megakaryoblasts resembling lymphoblasts.

**Figure 5:**
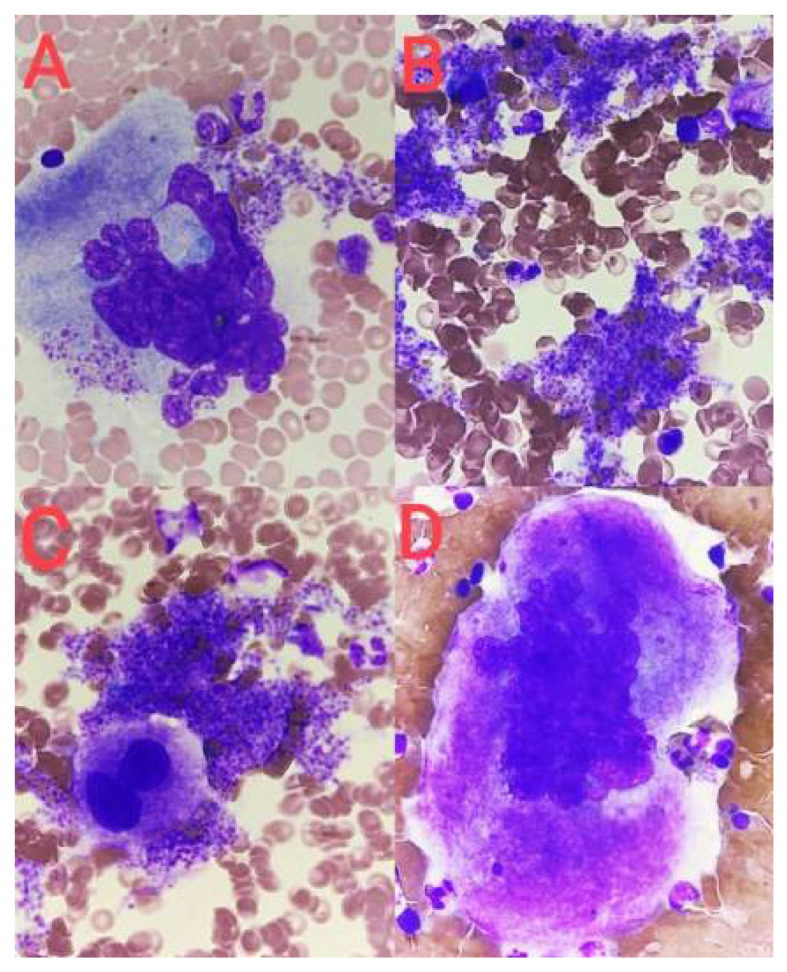
Megakaryocytes in chronic myeloproliferative neoplasms. (**A**) “Staghorn” megakaryocyte (essential thrombocythemia), (**B**) multiple platelet aggregations (essential thrombocythemia), (**C**) megakaryocyte showing increased platelet function, and (**D**) giant hypersegmented megakaryocyte (polycythemia vera).

**Figure 6:**
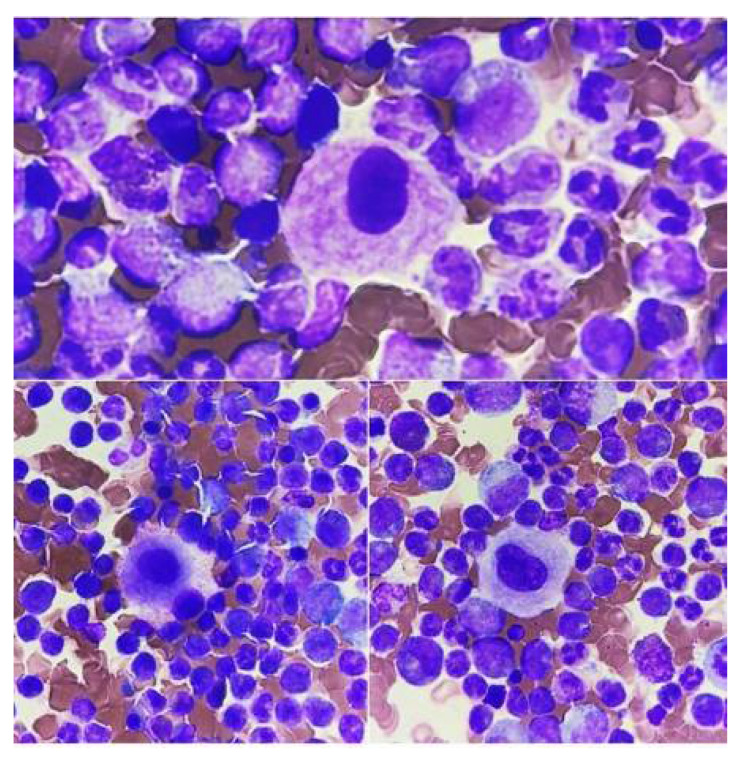
Micromegakaryocytes in chronic myeloid leukemia.

The quantitative and qualitative disorders of megakaryocytes in bone marrow cytological smears may be reactive (Figure 3) or primary (hematopoietic system neoplasms, Figure 4, Figure 5 and Figure 6). Both increased and decreased percentages of these cells are found in bone marrow cytological smears (Table 1). Qualitative and quantitative disorders are also characteristic for individual disease entities (Table 2).

**Figure 7:**
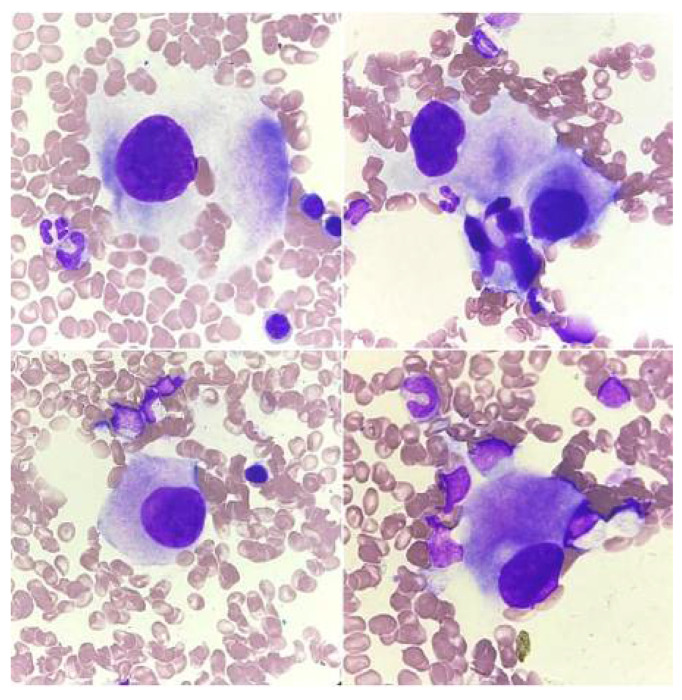
Megakaryocytes with hypolobulation of the nucleus in a patient with myelodysplastic neoplasm with del(5q) and with del(20q).

In the case of myelodysplastic neoplasms, detailed assessment of megakaryocyte morphology in cytological preparations is one of the most accurate methods for assessing the degree of dysplastic changes and has an advantage in this respect over histopathological examination. Moreover, certain morphological features of megakaryocytes correlate with specific cytogenetic abnormalities such as monosomy 7 or del(5q) (Figure 7). However, it should be noted that sometimes it is not possible to visualize megakaryocytes in bone marrow aspirate due to the unreliable quantity or poor quality of the obtained material for cytological assessment (so-called dry puncture, clotting of bone marrow aspirate in the aspiration needle, or admixture of peripheral blood), which may pose a major problem, especially regarding reliable quantitative assessment. In this case, a more accurate examination will be a histopathological assessment [10,12].

## 8. Dysmegakaryopoiesis

Dysmegakaryopoiesis is characterized by the presence of dysplastic megakaryocytes, which include micromegakaryocytes, forms with hypolobulation of the nucleus, multinucleated megakaryocytes, and megakaryocytes with hypogranulation of the cytoplasm [27].

## 9. Micromegakaryocytes

**Figure 8:**
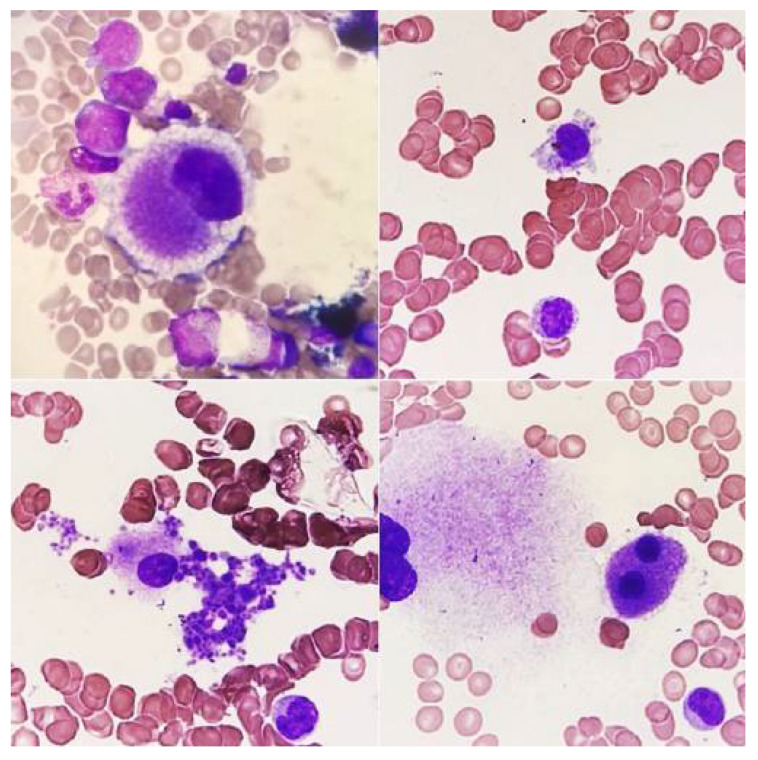
Micromegakaryocytes.

Micromegakaryocytes are also called “dwarf cells”. These are usually cells < 15 µm in size, although larger micromegakaryocytes are also observed but do not exceed 30–35 µm (most often, the cell diameter is smaller than twice the diameter of a neutrophil) [28,29]. The size of the nucleus is comparable to the nucleus of a myeloblast, with a compact chromatin structure. Most often, these are mononuclear cells, but there are also bi- or trinuclear forms, which are characterized by a high N/C ratio and the presence of a scanty, partially mature cytoplasm [10,11,12,30,31]. Micromegakaryocytes are shown in Figure 8.

## 10. Megakaryocytes

**Figure 9:**
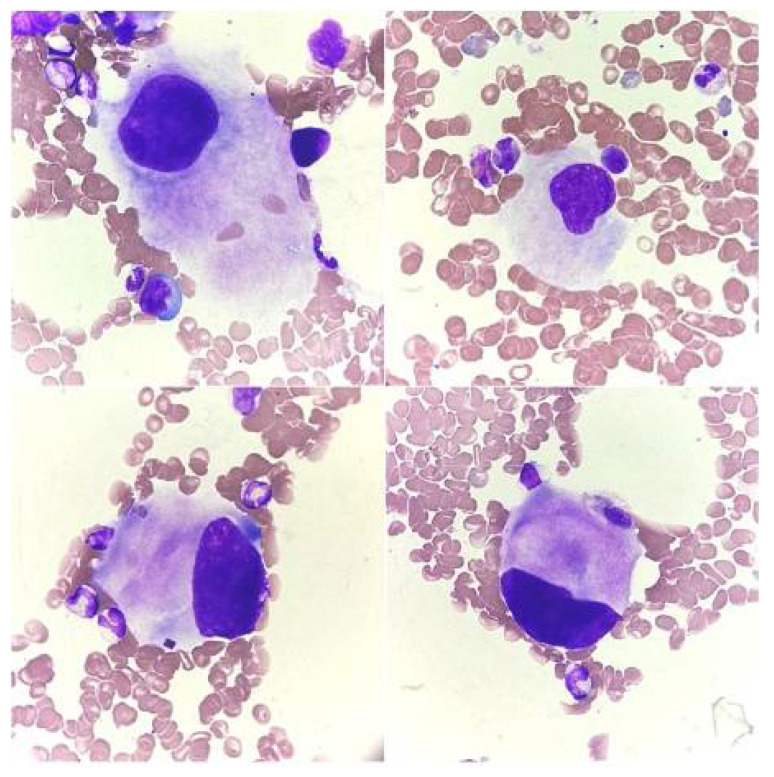
Megakaryocytes with hypolobulation of the nucleus.

Megakaryocytes with hypolobulation of the nucleus are usually the size of a normal megakaryocyte or are slightly smaller. They are characterized by the presence of a small, single-lobed, round nucleus; normal chromatin condensation; and abundant, mature cytoplasm (Figure 9). Most often, they demonstrate reduced platelet-forming function [10,12,30,31].

**Figure 10:**
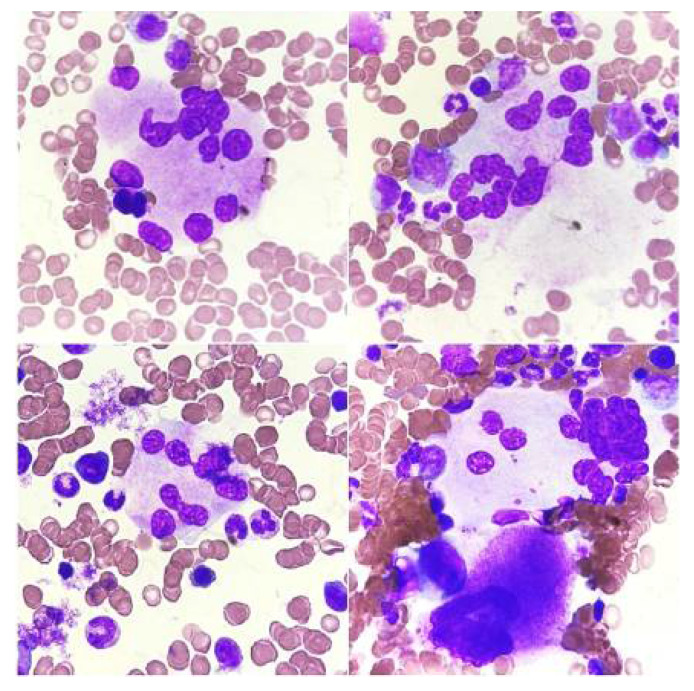
Dysplastic megakaryocyte with multiple separate nuclei.

Multinucleated megakaryocytes with distinctly separate nuclei are found in bone marrow cytology smears in patients with MDS or acute myeloid leukemia (AML) but may also be seen in healthy individuals [32]. They are shown in Figure 10. An increased number of multinucleated megakaryocytes as an isolated finding does not constitute a basis for the diagnosis of MDS [23,24].

## 11. Megakaryocytic Dysplasia

Megakaryocytic dysplasia is defined by morphological changes found in at least 10% of cells of this line (assessing at least 30 megakaryocytes). The most specific change for myelodysplastic neoplasms according to the 5th edition of the World Health Organization Classification (WHO 2022) is the presence of micromegakaryocytes [33]. Also, poor granulation of the megakaryocyte cytoplasm is common in MDS [30,31].

## 12. Dysmegakaryopoiesis in Healthy Donors

Features of dysmegakaryopoiesis have also been described in healthy donors. Bain et al. [34] described changes in megakaryopoiesis in the form of non-lobulated and multinucleated megakaryocytes. In all analyzed cases, the number of dysplastic cells was < 10%. Interestingly, a small percentage of non-lobulated or multinucleated megakaryocytes was quite frequent in healthy subjects with normal iron stores [34].

In the case of reactive changes (Figure 3), the most common findings in megakaryocytes are cytoplasmic maturation disorders, cytoplasmic vacuolation, megakaryocyte clusters, an increased number of young forms, increased platelet-forming activity, nuclear hypersegmentation, and naked nuclei [23].

## 13. Emperipolesis

**Figure 11:**
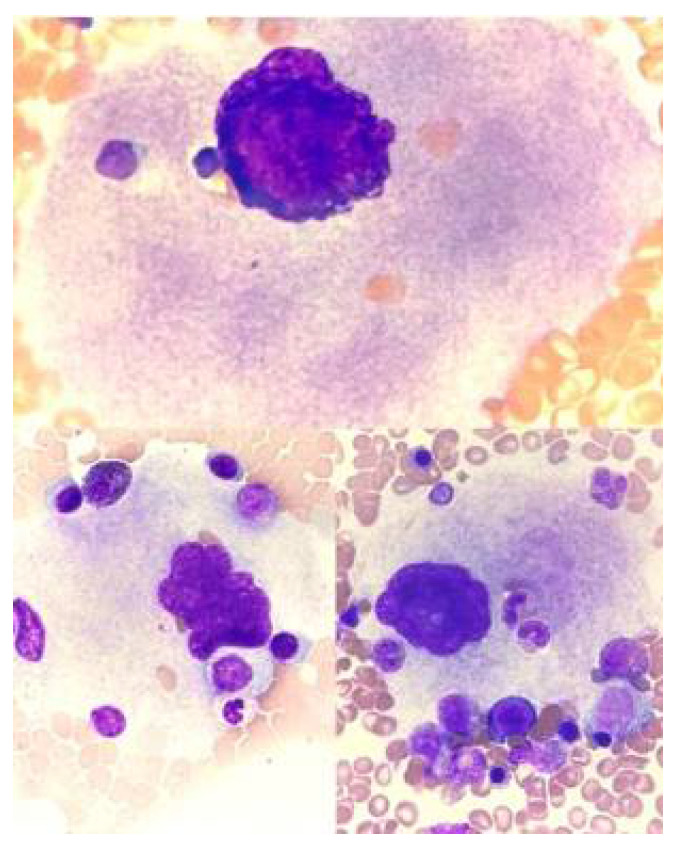
Emperipolesis in myeloproliferative neoplasms.

In bone marrow aspirate, megakaryocyte emperipolesis is sometimes observed. This is a phenomenon of the penetration and temporary “storage” of one cell within the cytoplasm of another cell. This cell does not undergo phagocytosis and can leave the cytoplasm unchanged. Emperipolesis involving megakaryocytes is observed in normal bone marrow and in pathological conditions (most often in myeloproliferative neoplasms). The cell engulfed by the megakaryocyte cytoplasm can be, in principle, a cell of any lineage and at any stage of maturation [23,35,36]. Emperipolesis is illustrated in Figure 11.

## 14. Basic Principles of Assessing Megakaryocytes in Bone Marrow Aspirate

Megakaryocytes are searched for using the lowest magnification of the lens (10×), also examining the edges and ends of the preparations. Due to their large size, most megakaryocytes remain inside the marrow nodules; therefore, the assessment performed in preparations made by crushing the nodules is considered more reliable. In accordance with the general recommendations, we do not include the total percentage formula of the myelogram but only use the terms none, single, quite numerous, or numerous [23]. The myelogram result should indicate the occurrence of megakaryocyte clusters, the presence of naked nuclei and platelet clusters, and fragments of the megakaryocyte cytoplasm if their number is significantly increased [27]. Platelet formation in the encountered megakaryocytes should also be assessed. In the case of dysplastic changes (where possible), 30 megakaryocytes should be assessed. Dysplasia is expressed as the percentage of dysplastic cells in a given cell series and lists the current features of dysplasia [23]. Detailed features of the nucleus, cytoplasm, and possible morphological abnormalities of megakaryocytes are assessed under 40× or 100× magnification [23]. However, fibrosis of the bone marrow, topographical alterations, and pattern of bone marrow involvement can be only assessed during histopathological evaluation [37]. Also, in cases of dry tap/scanty material, trepanobiotate evaluation is necessary [38]. Bone marrow examination is critical for diagnosing hematological malignancies and hematological diseases. In most cases, it cannot be replaced with other tests [39]. The application of neural networks and AI shows promise for the automation and standardization of bone marrow smear examination [40,41].

## 15. Differentiation of Megakaryocytes from Cells with Similar Morphology

**Figure 12:**
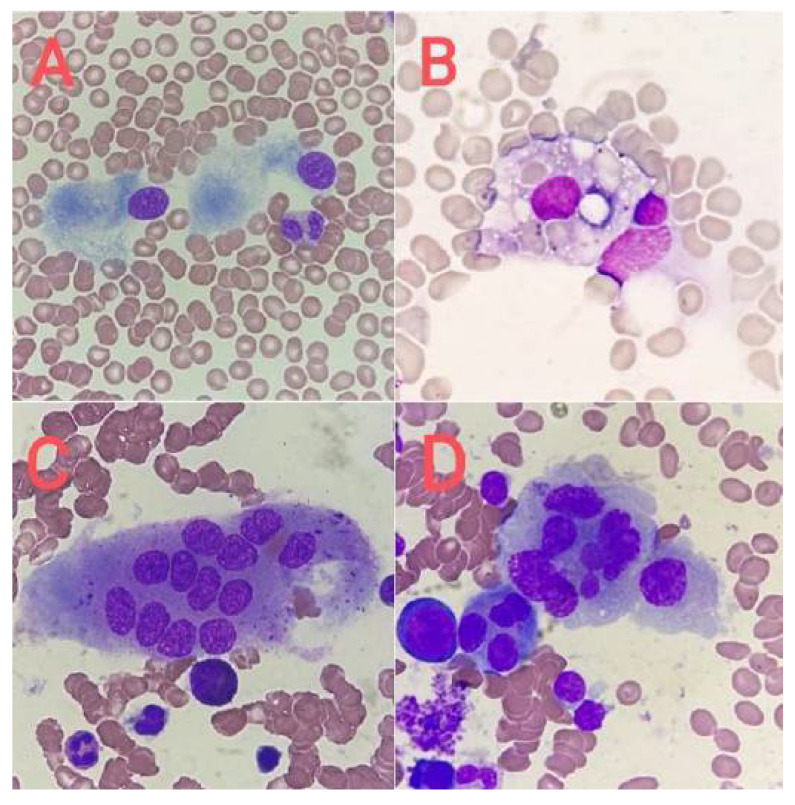
Cells morphologically resembling megakaryocytes. (**A**) Osteoblasts, (**B**) macrophage with hemophagocytosis, (**C**) osteoclast, and (**D**) atypical plasma cell.

In the bone marrow, cells that resemble megakaryocytes in their morphology can be found. To avoid the wrong classification of cells, it is important to know the morphology of both normal and dysplastic/atypical megakaryocytes and to have experience in assessing cytological preparations of bone marrow. The cells most often causing misclassification are as follows:

1. Osteoblasts (Figure 12A) incorrectly classified as megakaryocytes with hypolobulation of the nucleus;
2. Osteoclasts (Figure 12C) classified as dysplastic megakaryocytes with separated nuclear lobes;
3. Multinucleated, atypical plasma cells (Figure 12D) classified as dysplastic megakaryocytes;
4. Macrophages with hemophagocytosis (Figure 12B) described as the phenomenon of emperipolesis;
5. Metastatic cells or myeloblasts counted as megakaryoblasts [12].

## 16. Conclusions

From the everyday clinical practice point of view, it is extremely important to quickly obtain the results of additional tests that will allow for planning further stages of diagnostics. Cytological evaluation of the bone marrow is a simple method that provides information important to the clinician within a maximum of several hours. Knowledge of the morphology of normal megakaryocytes and their qualitative and quantitative changes allows for precise direction of the further diagnostic process. This translates into faster diagnosis of the disease. Microscopic evaluation of megakaryocytes in bone marrow aspirate, performed by an experienced diagnostician/hematologist, is still one of the most accurate methods for detecting even minor abnormalities and often exceeds histopathological evaluation in this respect. In the context of new drugs introduced to the therapy of hematological diseases, knowledge about the morphology, as well as qualitative and quantitative disorders of megakaryocytes during the course of treatment is still insufficient. Further research is necessary in this area.

## Figures and Tables

**Table 1:** Reasons for quantitative changes in normal and atypical megakaryocytes in bone marrow aspirate [10,12,25].

Decreased Megakaryocyte Count	Increased Megakaryocyte Count
Acute leukemias Advanced stages of PV and PMF Cancer metastases to bone marrow Viral infections (e.g., HIV, HCV, parvovirus B19, CMV, influenza virus, arboviruses, *Filioviridae*, EBV, HCV, and SARS-CoV-2) Aplastic anemia Treatment (e.g., chemotherapy and radiotherapy) Myelodysplastic neoplasms (e.g., MDS-h) Megaloblastic anemia Congenital amegakaryocytosis	Chronic myeloproliferative neoplasms Acute megakaryoblastic leukemia Immune thrombocytopenia Thrombotic thrombocytopenic purpura Hypersplenism Myelodysplastic neoplasms (e.g., MDS-5q) Blood loss

Abbreviations: PV—polycythemia vera, PMF—primary myelofibrosis, HIV—human immunodeficiency virus, MDS-h—hypoplastic myelodysplastic neoplasm, MDS-5q—myelodysplastic neoplasm with isolated del(5q), CMV—cytomegalovirus, and EBV—Epstein–Baar virus, HCV—hepatitis C virus.

**Table 2:** Features of megakaryocytes in individual disease entities [10,26].

Disease Entity	Morphological Features
Thrombotic thrombocytopenic purpura (TTP)	Increased number of normal megakaryocytes, often younger forms
Primary immune thrombocytopenia (ITP)	Increased number of megakaryocytes, often younger forms; numerous giant megakaryocytes and with hypolobulation of the nucleus; present clusters of megakaryocytes
Acquired hemolytic anemia	Small clusters of megakaryocytes with normal morphology
Iron deficiency anemia (IDA)	Normal megakaryocytes, sometimes increased platelet function
Megaloblastic anemia	Often reduced number of megakaryocytes; hypersegmentation of the nucleus; reduced granularity of the cytoplasm; present fragments of cytoplasm; naked nuclei of megakaryocytes
Chronic myeloid leukemia (CML)	Usually increased number of megakaryocytes; micromegakaryocytes present; intermediate N/C ratio
Essential thrombocythemia (ET)	Increased number of megakaryocytes; present giant forms with hypersegmented nucleus and abundant cytoplasm; present clusters of megakaryocytes, often forms with antler-shaped nuclei, so-called “staghorn”; numerous platelet clusters and cytoplasmic fragments, often megakaryocytes surrounded by platelet shoals; emperipolesis
Polycythemia vera (PV)	Increased number of megakaryocytes, distinct polymorphism (present forms of normal size and giant hypersegmented forms with abundant cytoplasm, less frequently megakaryocytes with hypolobulation of the nucleus, and blurred chromatin structure, so-called “cloud-like”)
Primary myelofibrosis (prefibrotic phase)	Megakaryocyte proliferation and atypia; small and large forms present with abnormal N/C ratio and hyperchromatic; megakaryocytes form dense clusters
Myelodysplastic neoplasms	Megakaryocyte count decreased, normal, or increased; micromegakaryocytes present, megakaryocytes with multiple separated nuclear lobes or nuclear hypolobulation; abnormal maturation of the nucleus in relation to the cytoplasm; cytoplasmic hypogranulation MDS with monosomy 7 (micromegakaryocytes) MDS with del(5q) (megakaryocytes with nuclear hypolobulation)
Acute megakaryoblastic leukemia	Blast cells with a high N/C ratio, tending to form clusters, which may suggest metastatic cells; morphologically, we distinguish megakaryoblasts resembling lymphoblasts, megakaryoblasts with basophilic cytoplasm forming small projections, and megakaryoblasts with cytoplasm in the form of long projections

Abbreviations: TTP—thrombotic thrombocytopenic purpura, ITP—immune thrombocytopenic purpura, IDA—iron deficiency anemia, CML—chronic myelogenous leukemia ET—essential thrombocythemia, and PV—polycythemia vera.

## Data Availability

No new data were created or analyzed in this study.
